# Effect of Post-Cam Design for Normal Knee Joint Kinematic, Ligament, and Quadriceps Force in Patient-Specific Posterior-Stabilized Total Knee Arthroplasty by Using Finite Element Analysis

**DOI:** 10.1155/2018/2438980

**Published:** 2018-09-19

**Authors:** Yong-Gon Koh, Juhyun Son, Oh-Ryong Kwon, Sae Kwang Kwon, Kyoung-Tak Kang

**Affiliations:** ^1^Joint Reconstruction Center, Department of Orthopaedic Surgery, Yonsei Sarang Hospital, 10 Hyoryeong-ro, Seocho-gu, Seoul 06698, Republic of Korea; ^2^Department of Mechanical Engineering, Yonsei University, 50 Yonsei-ro, Seodaemun-gu, Seoul 03722, Republic of Korea

## Abstract

The purpose of this study is to investigate post-cam design via finite element analysis to evaluate the most normal-like knee mechanics. We developed five different three-dimensional computational models of customized posterior-stabilized (PS) total knee arthroplasty (TKA) involving identical surfaces with the exception of the post-cam geometry. They include flat-and-flat, curve-and-curve (concave), curve-and-curve (concave and convex), helical, and asymmetrical post-cam designs. We compared the kinematics, collateral ligament force, and quadriceps force in the customized PS-TKA with five different post-cam designs and conventional PS-TKA to those of a normal knee under deep-knee-bend conditions. The results indicated that femoral rollback in curve-and-curve (concave) post-cam design exhibited the most normal-like knee kinematics, although the internal rotation was the closest to that of a normal knee in the helical post-cam design. The curve-and-curve (concave) post-cam design showed a femoral rollback of 4.4 mm less than the normal knee, and the helical post-cam design showed an internal rotation of 5.6° less than the normal knee. Lateral collateral ligament and quadriceps forces in curve-and-curve (concave) post-cam design, and medial collateral ligament forces in helical post-cam design were the closest to that of a normal knee. The curve-and-curve (concave) post-cam design showed 20% greater lateral collateral ligament force than normal knee, and helical post-cam design showed medial collateral ligament force 14% greater than normal knee. The results revealed the variation in each design that provided the most normal-like biomechanical effect. The present biomechanical data are expected to provide useful information to improve post-cam design to restore normal-like knee mechanics in customized PS-TKA.

## 1. Introduction

End-stage knee osteoarthritis is often treated with total knee arthroplasty (TKA) [1]. However, conventional TKA geometries are based on anthropometric data that accommodate the anatomic variations of most knees [2–4]. An adequate fit is achieved in most cases. However, a mismatch exists at some frequencies, which can theoretically impact the clinical outcomes. Anteroposterior (AP) oversizing of the femoral component alters the flexion gap, leading to tightness or anterior overstuffing, which increases the risk of patellofemoral (PF) symptoms postoperatively [5, 6]. Medial or lateral overhang of either the femoral or tibial component may cause soft-tissue impingement [2, 7]. To overcome these problems, the use of customized TKAs has been suggested [8]. Thus, customized TKA techniques have been introduced to mimic these conventional TKA size mismatch problems and native anatomy mimetic [9–12]. In general, magnetic resonance imaging (MRI) or computer tomography (CT) scans can be used to provide data on manufacturing a customized TKA and instrumentation system.

Specifically, patient-specific instrumentation involves better preoperative planning [13–16]. Hence, both the cutting jigs and the implant are specifically designed for the patient in customized TKA [13]. The preoperative imaging studies used for manufacturing custom implants are the same as those used for manufacturing jigs with native femoral characteristics such as intercondylar notch distance, the “J” curve, the condylar offset, anteroposterior and mediolateral width, and native tibial bone size and coverage [13]. Thus, the aforementioned advantages are associated with customized TKA. Unicompartmental, bicompartmental, cruciate-retaining (CR) TKA, and posterior-stabilized (PS) TKA have been recently introduced as variations of customized TKA [9]. The customized TKA has improved in biomechanical and clinical results [17–22]. However, these studies are limited to CR customized TKA. Also, the design of post-cam is important to maintain normal knee biomechanics in customized PS-TKA.

Conventional PS-TKA exhibits satisfactory long-term survival rates and good functional performance [23–25]. The post-cam mechanism in PS prostheses plays an important role in TKA. The post-cam mechanism in PS-TKA prevents posterior subluxation of the tibia in flexion and restores femoral rollback [26, 27], indicating that this mechanism is important in knee kinematics. Consequently, it is important to understand the effect of post-cam design on knee motion. The features of post-cam design are typically categorized into flat-on-flat or curve-on-curve surfaces in the contemporary PS-TKA [28]. A previous study evaluated the contact pressure of the post-cam mechanism in different TKA designs and suggested that high contact pressures exist at different post designs with tibial rotation. Thus, the post-cam design may be altered to provide a larger contact area with reduced edge loading [29]. Lin et al. investigated tibiofemoral (TF) motion of various post-cam designs during high knee flexion [28]. They observed that the curve-on-curve design exhibited internal tibial rotation, which corresponded to the post-cam engagement to extreme flexion [28]. However, they only investigated curve-and-curve and flat-and-flat designs. As mentioned earlier, post-cam design in PS-TKA is an important factor affecting knee joint biomechanics. Additionally, extant studies do not consider the preservation of normal knee mechanics for post-cam design in customized PS-TKA.

The purpose of the present study was to clarify the preservation of normal knee mechanics for five post-cam designs in customized PS-TKA and conventional PS-TKA. Customized PS-TKA is categorized into (1) flat-and-flat, (2) curve-and-curve (concave), (3) curve-and-curve (concave and convex), (4) helical, and (5) asymmetrical post-cam designs. A conventional PS-TKA with curve-and-curve post-cam design was also analyzed for comparison to the customized designs. The kinematics, collateral ligament force, and quadriceps force are investigated for these five post-cam designs in customized PS-TKA and conventional PS-TKA by using three-dimensional (3D) finite element (FE) analysis under deep-knee-bend activity.

## 2. Material and Methods

### 2.1. Design of Post-Cam and Customized PS-TKA

**Figure 1:**
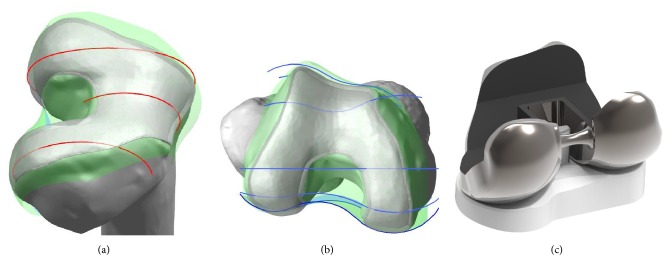
Development of customized PS-TKA: (a) three patient-specific “J” curves in sagittal planes; (b) patient's anatomic curves in coronal planes; (c) femoral component and tibial insert of customized PS-TKA.

Customized PS-TKA was developed using a 3D knee joint FE model used in a previous study [17, 30, 31]. A 3D knee joint model was developed from CT and MRI data followed by 3D reconstruction by using Mimics 17.0 (Materialize, Leuven, Belgium). Based on the dimensions of the femur and tibia, devices corresponding to conventional PS-TKA (Genesis II Total Knee System; Smith & Nephew, Inc., Memphis, TN, USA) sizes 7 and 5-6 were selected for the femoral component and tibial insert, respectively. In contrast, the customized PS-TKA femoral component was AP 74 mm and mediolateral (ML) 84 mm. The tibial insert was AP 57 mm and ML 78 mm. Planes were introduced by intersection of the condyles in both the sagittal and coronal planes. Intersection curves were used to extract the articulating surface geometry in both planes. The three patient-specific “J” curves for the trochlear grooves and the medial and lateral condyles from the normal articular anatomy of patients were developed using the Unigraphics NX software (Version 7.0; Siemens PLM Software, Torrance, CA, USA) (Figure 1). The customized femoral component uses these patient-specific differences and is designed by using the coronal offset of a patient [9–12, 32, 33]. The coronal offset is defined as the height difference between the medial and lateral femoral condyles in the coronal extension plane. With respect to the tibial insert, the profile of the patient's tibia defines the geometry of the tibial implant (Figure 1). Generally, articular geometry in customized tibial insert design is derived from the femoral component. The medial insert geometry is slightly more conforming when compared with that of the lateral insert [9, 12]. The coronal geometry provides a broad radius for both condyles and thereby employs the round-on-round principle associated with a reduction in contact stress [9, 12].

**Figure 2:**
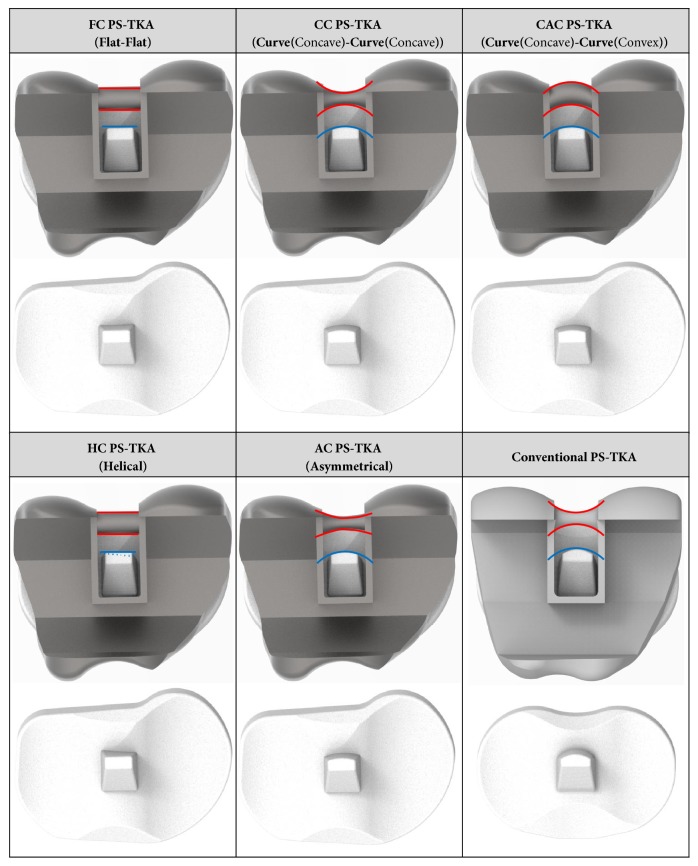
Customized PS-TKAs with five different post-cam design and conventional PS-TKA.

We developed five different 3D models for customized PS-TKA with identical surfaces with the exception of the post-cam geometries including the intercondylar notch of the femoral component. The post-cam design for customized PS-TKA was categorized into flat-and-flat customized PS-TKA (FC PS-TKA), curve-and-curve (cam: concave) customized PS-TKA (CC PS-TKA), curve-and-curve customized PS-TKA (cam: concave and convex) (CAC PS-TKA), helical customized PS-TKA (HC PS-TKA), and asymmetrical customized PS-TKA (AC PS-KTA) (Figure 2).

We developed a customized PS-TKA by applying the same ratio of post and cam positions of conventional PS-TKA. We controlled the post anterior-posterior position, post size (height, width and depth), and cam position (distance from the posterior edge and height above the joint line) to solely investigate the effect of post-cam design.

### 2.2. Development of Normal Knee Fe Model

**Figure 3:**
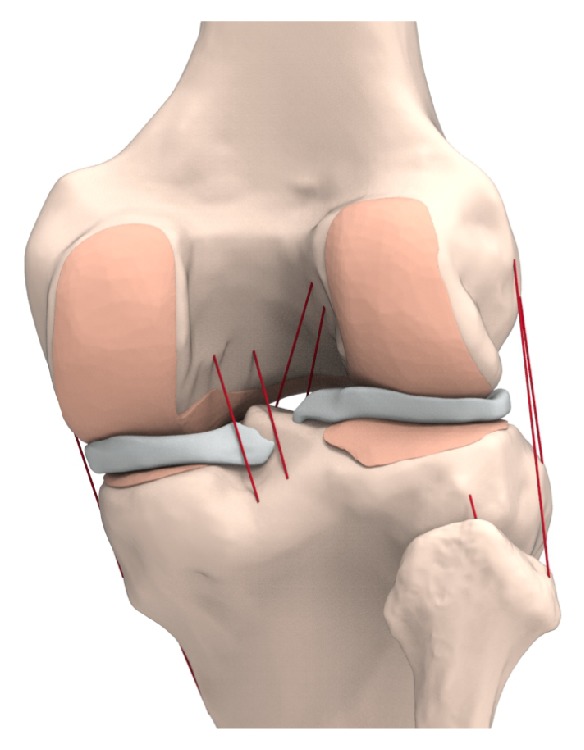
Validated 3D FE models used in this study.

For this study, a validated subject-specific FE model was used. The procedure of development of the existing validated normal knee FE model is briefly described below [17, 30, 31, 34, 35]. The FE model of a normal knee joint was developed by using data from the medical images of a healthy 37-year-old male subject. The model includes bony structures of the lower extremity in addition to soft-tissue details of PF and TF aspects of the knee joint. The model includes major ligaments, articular cartilage, and menisci (Figure 3). The bony structures were modeled as rigid bodies [27]. Cartilage was modeled as an isotropic linear elastic material. Menisci were modeled as transversely isotropic linear elastic with different mechanical properties in circumferential, axial, and radial directions [34]. All ligament bundles were modeled as nonlinear springs with material properties obtained from a published report [35–37]. The ligaments were simulated as nonlinear force elements with their parabolic and linear equations as follows:* ε* < 0,* f*(*ε*) = 0; if 0 ≤* ε* ≤ 2*ε*_1_,* f*(*ε*) =* kε*^2^/4*ε*_*1*_; and if *ε*> 2*ε*_1_,* f*(*ε*) =* k*(*ε*-*ε*_*1*_), where* f* is the tension of the ligament,* ε* is the ligament strain, and* k* is the stiffness coefficient of each ligament. The linear range threshold was specified as *ε*_1_=0.03. The interfaces between the articular cartilage and bones were assumed to be fully bonded. Six pairs of TF contacts between the femoral cartilage and meniscus, meniscus and tibial cartilage, and femoral cartilage and tibial cartilage were modeled for both the medial and lateral sides [34].

### 2.3. Development of Different Post-Cam Designs in the Customized TKA and Conventional TKA

**Figure 4:**
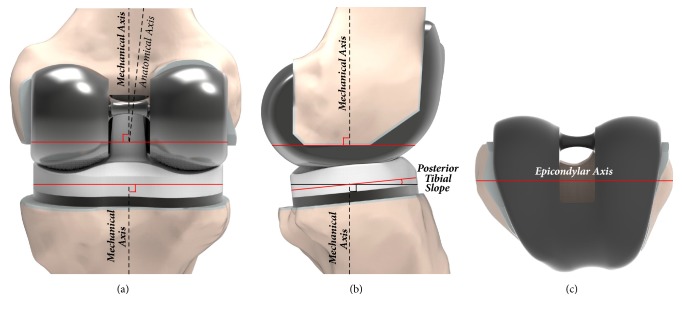
Surgical method used to develop the customized and conventional PS-TKA models: (a) coronal plane; (b) sagittal plane; (c) transverse plane.

Customized and conventional TKA models were implanted as described below. In aligning the components in the coronal plane, the femoral component was set perpendicular to the mechanical axis that connected the center of the knee and the center of the femoral head, and the tibial component was set perpendicular to the mechanical axis that connected the center of the knee and the center of the ankle joint. The neutral rotational alignments of the femoral and tibial components were positioned in line with the femoral epicondylar axis and the tibial anteroposterior axis, respectively (Figure 4). Contact conditions were applied between the TF and PF in TKA. The coefficient of friction between the polyethylene and metal materials was assumed to be 0.04 to ensure consistency with previous explicit FE models [38]. The materials employed for the femoral component, polyethylene insert, and tibial baseplate have been described in previous studies [17, 38].

### 2.4. Boundary and Loading Conditions

There are four types of loading conditions corresponding to the loads used in the experiments in the study for model validation and predictions for clinically relevant under deep-knee-bend loading conditions. The first and second loading conditions are the intact model validation, the third is the validation of the TKA model, and the fourth is the predictions for clinically relevant scenarios.

In the first loading condition, 150 N was applied to the tibia with 30° and 90° flexion in the FE knee joint to measure the anterior tibial translation and posterior tibial translation, respectively [35]. Additionally, a second axial loading of 1,150 N was applied to the model to obtain the contact pressures to facilitate a comparison with a published study on the knee joint FE analysis [39]. A conservative ankle force of 50 N and a hamstring force of 10 N were constantly exerted with a linearly increasing force, and a maximum force of approximately 600 N at 90° flexion of the quadriceps actuators for the TKA model was applied under the third loading condition [40]. The fourth loading conditions included deep-knee-bend loading applied to evaluate the effects of post-cam design of customized TKA on the generation of normal knee mechanics [21, 41–44]. A computational analysis was performed with anterior-posterior force applied to the femur with respect to the compressive load applied to the hip with femoral internal-external rotation constrained, medial-lateral translation free, and knee flexion determined by a combination of vertical hip and quadriceps load, creating a six-degree-of-freedom TF joint [41–43]. A proportional-integral-derivative control was incorporated into the computational model to control the quadriceps in a manner similar to that in a previous experiment [45]. A control system was used to evaluate the instantaneous quadriceps muscle displacement required to match a target flexion profile, which was the same as that used in the experiment. Internal-external and varus-valgus torques were applied to the tibia, with the remaining tibial degree of freedoms constrained [41–43].

The FE model was analyzed by using the ABAQUS software (version 6.11; Somalia, Providence, RI, USA). We investigated kinematics, collateral ligament force, and quadriceps force to evaluate the manner in which close normal knee mechanics are restored in customized TKA for five different post-cam designs when compared with those in conventional TKA. A three-cylindrical knee joint model was developed with six degrees of freedom for the relative kinematics of the TF and PF articulations [46]. Embedded coordinate frames in the femur, tibia, and patella were considered using nodes, and their positions were evaluated under the loading conditions.

## 3. Results

### 3.1. Validation of Normal Knee and Conventional TKA FE Model

Based on an already validated and published normal knee finite element model, the model used in this study briefly explained validation results [35, 39]. In order to validate the FE model, it was compared with the results from the experiment with FE subject. In the loading condition with 30° flexion, the anterior tibial translation was 2.83 mm in the experiment and 2.54 mm in the FE model, and the posterior tibial translation was 2.12 mm in the experiment and 2.18 mm in the FE model for validation. Similarly, with respect to the 90° flexion, the anterior tibial translation was 3.32 mm in the experiment and 3.09 mm in the FE model, and the posterior tibial translation was 2.64 mm in the experiment and 2.71 mm in the FE model [35]. Additionally, the results were also compared with previous FE results for model validation. Contact pressures corresponding to 3.1 MPa and 1.53 MPa were observed on the medial and lateral meniscus, respectively, under an axial load of 1,150 N. Both were within 4% of the contact pressures corresponding to 2.9 MPa and 1.45 MPa as reported by Peña et al. [39]. These minor differences could be caused by variations in the geometry such as the thickness of the cartilage and meniscus between different studies.

**Figure 5:**
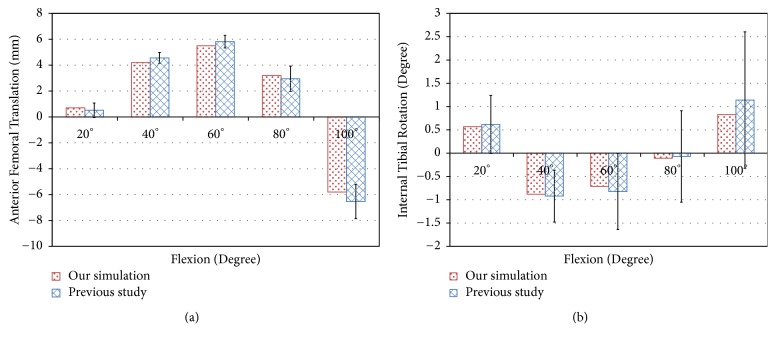
Comparison of kinematics with previous study for validation of TKA model: (a) anterior femoral translation; (b) internal tibial rotation.

The conventional TKA FE model was compared with previous experimental data for validation. The FE model for the femur was translated by 0.7 mm, 4.2 mm, 5.5 mm, 3.2 mm, and -5.8 mm in anterior direction at 20°, 40°, 60°, 80°, and 100° flexion, respectively (Figure 5(a)). In addition, The FE model for the tibia was internally rotated by 0.57°, -0.88°, -0.71°, -0.11°, and 0.83° under 20°, 40°, 60°, 80°, and 100° flexion, respectively (Figure 5(b)). The data of our simulation study were within ±1 standard deviation of the average reported in the previous study using the same boundary conditions and TKA design [40].

### 3.2. Comparison of Kinematics between Customized PS-TKA and Conventional PS-TKA with Respect to Post-Cam Design

**Figure 6:**
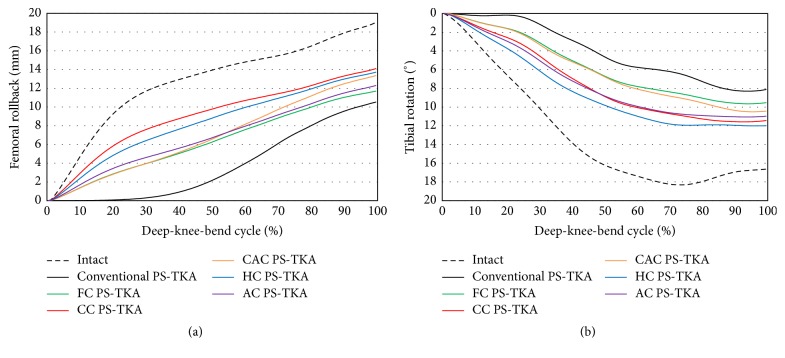
Comparison of (a) femoral rollback and (b) tibial rotation between conventional PS-TKA and five different post-cam designs of customized PS-TKA under deep-knee-bend activity.

There were differences in the results of the post-cam design that restored normal knee kinematics in femoral rollback and internal rotation (Figure 6). With respect to both femoral rollback and internal rotation, conventional PS-TKA exhibited the worst normal-like kinematics. Customized and conventional PS-TKA exhibited reduced femoral rollback when compared with normal knee. However, with respect to the femoral rollback, CC PS-TKA exhibited the most normal-like rollback pattern. CC PS-TKA showed 4.4 mm less femoral rollback than normal knee. Additionally, customized and conventional PS-TKA displayed reduced internal rotation when compared with that of the normal knee. The conventional PS-TKA did not exhibit the characteristic screw home motion between 0° and 30° flexion. With a further increase in knee flexion, either a slow increase in internal tibial rotation was exhibited or a near constant rotational position was maintained in conventional PS-TKA. However, all customized PS-TKA displayed screw home mechanism in flexion. Specifically, HC PS-TKA displayed the most normal-like internal rotation. HC PS-TKA showed 5.6° less internal rotation than normal knee during deep-knee-bend activity.

### 3.3. Comparison of Collateral Ligament Force and Quadriceps Force in Customized and Conventional PS-TKA for Different Post-Cam Designs

**Figure 7:**
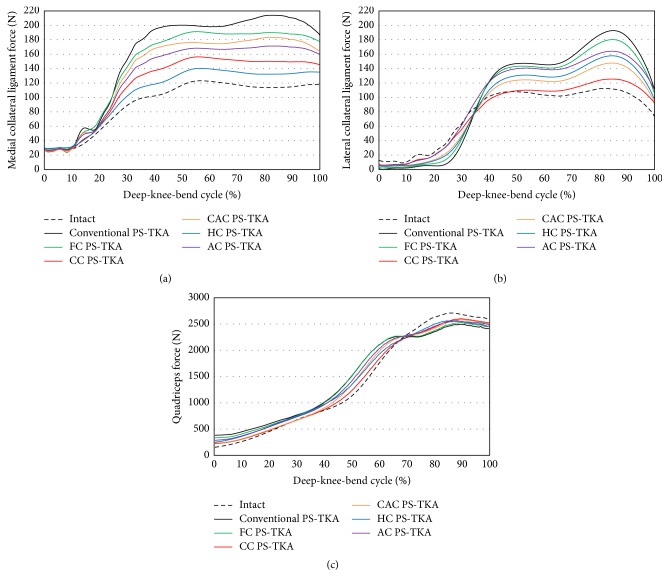
Comparison of (a) medial collateral ligament force, (b) lateral collateral ligament force, and (c) quadriceps force between conventional PS-TKA and five different post-cam designs of customized PS-TKA under deep-knee-bend activity.

All the customized and conventional TKA designs exhibited higher collateral ligament force when compared with that observed with respect to the normal knee (Figure 7). The conventional PS-TKA displayed the highest increase relative to the normal knee design. With respect to the medial and lateral collateral ligament, HC and CC PS-TKA displayed the most normal-like ligament forces, respectively. In all the TKA designs, higher and lower quadriceps forces were required in low and high flexions, respectively, when compared with those in the case of a normal knee. The CC PS-TKA exhibited the most normal-like quadriceps force.

## 4. Discussion

The most important finding of this study is that different post-cam designs of customized PS-TKA exhibited differences in the restoration of normal knee mechanics. In addition, the customized PS-TKA models could not restore normal knee mechanics. However, the results exhibited by customized PS-TKA were better than those exhibited by conventional PS-TKA.

Recent studies that focused on customized TKA are associated with good clinical reports, although most of them were limited to CR-TKA [18–20, 22]. Previous studies did not focus on post-cam design in customized PS-TKA. It has also been demonstrated in the importance of post-cam design for conventional TKA. In addition, the restoration of normal knee kinematics after TKA is an extremely important topic. Numerous studies investigated the effects of customized TKA on knee kinematics to restore normal knee kinematics although they did not consider the post-cam mechanism [17–22]. Additionally, several studies focused on the post-cam mechanism in conventional PS-TKA albeit with respect to contact stress [47–51]. The post-cam mechanism is important in TF motion during high knee flexion [52]. Therefore, the purpose of this study was to investigate post-cam designs in customized PS-TKA with respect to restoration of normal knee mechanics. The intact knee model was validated, and the results indicated good consistency with previous experimental data in terms of kinematics and contact area as demonstrated by the FE analysis with an identical subject [30, 34, 35]. Additionally, the conventional PS-TKA model was validated using experimental and kinematics data [40]. Therefore, the TKA model developed in this study is considered reasonable. The computational simulation in this study involved a single subject, and this is advantageous because it is possible to determine the effects of post-cam design for customized PS-TKA with an identical subject without the effect of variables such as weight, height, bony geometry, ligament properties, and component size [53].

The results in the present study indicate that there are differences in the preservation of normal knee kinematics in each post-cam design. With respect to femoral rollback, the CC PS-TKA exhibited the most normal-like kinematics. Several previous studies reported that the biomechanics exhibited in the curve-and-curve design were better than those exhibited in the flat-and-flat design [28, 47–49]. A recent study demonstrated that the circle cam and convex post provided the optimal femoral rollback effect leading to the highest amount of knee flexion [54]. The aforementioned results are in accordance with the results obtained in the present study. However, with respect to internal rotation, the HC PS-TKA exhibited the most normal kinematics. A previous study revealed that the internal tibial rotation of the curve-on-curve design exceeded that of the flat-and-flat design [28]. The aforementioned trend was also observed in our study, although this effect was lower than that of the HC PS-TKA. Internal tibial rotation was coupled with posterior femoral translation during flexion, and it is important in knee motion.

One of the most controversial recent issues in TKA involves the achievement of deep knee flexion. Thus, kinematic analysis of deep knee flexion was also considered for TKA implanted knees in several studies [55–57]. Nakamura et al. reported a mean internal tibial rotation of 17° in deep flexion [57]. In contrast, only minimal internal rotation from mid-flexion to deep flexion was observed in previous studies [55, 56]. The computational analysis in the present study indicated a consistent internal tibial rotation with flexion and a further increase in internal rotation after 90° of flexion to the maximum knee flexion under deep-knee-bend activity. This characteristic feature in flexion kinematics is an apparent advantage of the helical post-cam design. The aforementioned trend was observed in the previous image analysis [58].

Interesting results were also observed in terms of the collateral ligament and quadriceps forces. The most normal-like outcomes were associated with HC PS-TKA for the medial collateral ligament force under deep-knee-bend activity. This is because the medial pivot was more allowable, and more internal rotation occurred in flexion owing to the screw home mechanism in post-cam design for HC PS-TKA. However, CC PS-TKA exhibited the most normal-like pattern in terms of the lateral collateral ligament force and quadriceps force.

Several previous studies reported that the curve-and-curve design exhibited better biomechanical effect than the flat-and-flat design [28, 49, 54]. A reduced tibial rotation occurred relative to knee flexion. For example, the tibia rotated externally relative to the femur during flexion. This indicated that a reverse torsion that originated from a rotational force was exerted on the tibia [28]. It was induced by medial impingement of post-cam on the tibia, leading to external tibial rotation during the post-cam interaction [28]. However, the post-cam designs could not restore normal knee mechanics even in customized PS-TKA. The main reason is the absence of anterior cruciate ligament (ACL). Recent computational studies supported the finding that bicruciate retaining TKA exhibited more normal-like knee kinematics [17, 59]. Furthermore, Zumbrunn et al. reported that the absence of ACL function is linked to abnormal kinematics and joint stability in patients with conventional TKA [60]. Moreover, ACL-substituting TKA could be a valuable option to overcome the limitations of conventional TKA, especially, when it is not possible to retain the native ACL [60]. Van Duren et al. also used image analysis and reported that bicruciate stabilized TKA did not exhibit any paradoxical anterior movement and sufficient posterior femoral roll back that corresponded to the engagement of the anterior and posterior post-cam mechanisms [61]. Therefore, the replacement of ACL function should be considered in restoring normal knee mechanics in customized PS-TKA.

In terms of biomechanical point of view, identifying the optimal design of post-cam should help manufactures customized the PS-TKA. In the present study, our results demonstrate that the biomechanical effect varies from customized TKA to post-cam design. Our results show that the curve-and-curve design of post-cam improved the best biomechanical effect. However, the design of location and size is not customized to respect each patient's unique “J” curves through the range of motion. Therefore, it is necessary to study not only the post-cam shape but also the location and size.

The current study involves four limitations. First, the five specific post-cam designs used in this study do not represent all the design features of contemporary TKA. Second, a deep-knee-bend simulation was performed although simulations related to more demanding activities (e.g., chair rising, sitting, stair climbing, and stair descending) are required in the future for a more reliable investigation. However, the simulation was performed under deep-knee-bend motion because it includes both a wide range of flexion-extension and a significant muscular endeavor around the knee joint. Third, implant kinematics and quadriceps force were evaluated by using computational simulations, and this does not fully represent an* in vivo* condition. Fourth, the anatomy for the customized PS design was based on, and virtually implanted in, only one subject. The use of subjects of various ages would improve the validity of the results because the validity is also dependent on the geometry of the knee joint. Most significantly, the time and computational cost associated with subject-specific FE model generation were not efficient. Future research will increase the number of subjects.

Despite the aforementioned limitations, this study provided insights into post-cam design for customized PS-TKA to restore normal knee mechanics. The strengths of computational simulation include the avoidance of individual difference, and the analyzed results have high repeatability [28].

In conclusion, post-cam design influences knee mechanics in customized PS-TKA. There are differences in the restoration pattern for normal knee mechanics for each post-cam design. However, all customized PS-TKA models did not perfectly preserve normal knee mechanics. As shown in the study, further design modifications to the customized TKA are required to achieve normal knee mechanics during deep-knee-bend activity. Additionally, it is necessary to consider the design for substituting ACL function.

## Data Availability

The data used to support the findings of this study are available from the corresponding author upon request.
